# Evolution of reproductive traits have no apparent life-history associated cost in populations of *Drosophila melanogaster* selected for cold shock resistance

**DOI:** 10.1186/s12862-021-01934-2

**Published:** 2021-12-06

**Authors:** Karan Singh, Ekta Kochar, Prakhar Gahlot, Karan Bhatt, Nagaraj Guru Prasad

**Affiliations:** 1 Present Address: Department of Cell Biology, NYU Grossman School of Medicine, 650 Medical Science Building, 550 First Ave, New York, NY 10016 USA; 2 Indian Institute of Science Education and Research Mohali, Knowledge City, Sector 81, SAS Nagar, PO Manauli, Mohali, Punjab 140306 India

**Keywords:** Adult mortality, Egg viability, Mating frequency, Life-history evolution, Lifetime fecundity, Longevity, Reproductive traits, Trade-off

## Abstract

**Background:**

In insect species like *Drosophila melanogaster*, evolution of increased resistance or evolution of particular traits under specific environmental conditions can lead to energy trade-offs with other crucial life-history traits. Adaptation to cold stress can, in principle, involve modification of reproductive traits and physiological responses. Reproductive traits carry a substantial cost; and therefore, the evolution of reproductive traits in response to cold stress could potentially lead to trade-offs with other life-history traits. We have successfully selected replicate populations of *Drosophila melanogaster* for increased resistance to cold shock for over 33 generations. In these populations, the ability to recover from cold shock, mate, and lay fertile eggs 24 h post cold shock is under selection. These populations have evolved a suite of reproductive traits including increased egg viability, male mating ability, and siring ability post cold shock. These populations also show elevated mating rate both with and without cold shock. In the present study, we quantified a suite of life-history related traits in these populations to assess if evolution of cold shock resistance in these populations comes at a cost of other life-history traits.

**Results:**

To assess life-history cost, we measured egg viability, mating frequency, longevity, lifetime fecundity, adult mortality, larva to adult development time, larvae to adults survival, and body weight in the cold shock selected populations and their controls under two treatments (a) post cold chock and (b) without cold shock. Twenty-four hours post cold shock, the selected population had significantly higher egg viability and mating frequency compared to control populations indicating that they have higher cold shock resistance. Selected populations had significantly longer pre-adult development time compared to their control populations. Females from the selected populations had higher body weight compared to their control populations. However, we did not find any significant difference between the selected and control populations in longevity, lifetime fecundity, adult mortality, larvae to adults survival, and male body weight under the cold chock or no cold shock treatments.

**Conclusions:**

These findings suggest that cold shock selected populations have evolved higher mating frequency and egg viability. However, there is no apparent life-history associated cost with the evolution of egg viability and reproductive performances under the cold stress condition.

**Supplementary Information:**

The online version contains supplementary material available at 10.1186/s12862-021-01934-2.

## Background

A number of ecological factors, including temperature, are known to vary across time and space, and as a result, organisms experience different types of environmental stresses during their lifespan. These environmental stresses can be major drivers of the evolution of the life-history of organisms in nature [1, 2].

Temperature is one of the fundamental ecological features that affects various life-history and related traits of insects such as development time, fecundity, male fertility, mating ability, motility, lifespan, and reproduction [3–17].

Organisms can respond to extreme temperatures in various ways, like changes in their behavioral patterns and physiology or life-history traits [1, 18, 19]. Resources used for coping with stress are unavailable for other functions under limited resource conditions, leading to trade-offs across important life-history traits such as somatic maintenance and reproduction [20]. For example, one important way in which organisms cope with immediate temperature changes (heat shock and cold shock) is by expressing heat shock proteins (*Hsps*). Expression of these proteins is extremely costly and affects reproduction [21]. Thus, investment in resisting temperature shock can lead to energy-based trade-offs with other important life-history traits [22–25].

Several studies have investigated the evolution of life-history traits in response to thermal variation. Widely distributed *D. melanogaster* being offers a great model to study the evolution of life-history traits in response to temperature variation across latitudes and altitudes. In general, a number of traits vary progressively across populations inhabiting various latitudes. Latitudinal clines have been found in a number of life-history traits such as development time, survivorship, larval competitive ability, fecundity, and body size [4, 26–30]. This pattern of results suggests that environmental differences are primarily driving life-history evolution in populations of *Drosophila* and that the populations are adapting to the local environment, possibly, including temperature, which is an important component of the environment.

Adaptation to the thermal environment can involve modifications of reproductive traits and physiological response in *D. melanogaster* and shown cold shock drastically affects egg viability in *D. melanogaster*. Therefore, it is essential to produce active gametes and mate to produce fertile eggs post cold shock [14]. Accordingly, the cold shock selected populations mate more frequently than their control populations post cold shock [12–14]. Moreover, it has been known that courtship and mating carry a substantial cost to both males and females [31]. Thus, the costs of the evolution of cold shock resistance are expected to be substantial*.* So far, very few experimental studies have assessed the evolution of life-history traits in response to selection for cold stress tolerance [7, 24, 32–35]. For example Anderson et al. [35] reported increased females fecundity and decreased males longevity in populations of *D. melanogaster* selected for rapid chill-coma recovery. MacMillan et al. [24] documented reduced longevity in females (but not in males) in populations selected for increased resistance to cold shock. To the best of our knowledge, this is the first study to investigate the underlying life-history cost to increased reproductive performance and egg viability to cold stress, we assayed various life-history (longevity, lifetime fecundity, mating frequency, egg viability, and adults mortality) and related traits such as larva to adult development time, larvae to adults survival, and body weight in the cold shock selected populations (FSB) and their control populations (FCB).

### Results

#### Experiment 1: egg viability and mating frequency

After 24 generations of selection, we first wanted to investigate that if there was a primary response to selection for cold shock resistance. The effects of cold shock selection on egg viability and mating frequency were studied again to see whether the previously observed response to cold selection persisted and to evaluate the potential trade-offs associated with the evolution of cold resistance [14]. In our selection regime, the ability to recover from cold shock, mate and lay fertile eggs 36 h post cold shock is under selection. Therefore, we assayed the evolution of cold shock resistance in terms of (a) egg viability 24–30 h post cold shock and (b) mating frequency over the first 36 h post cold shock.

**Fig. 1:**
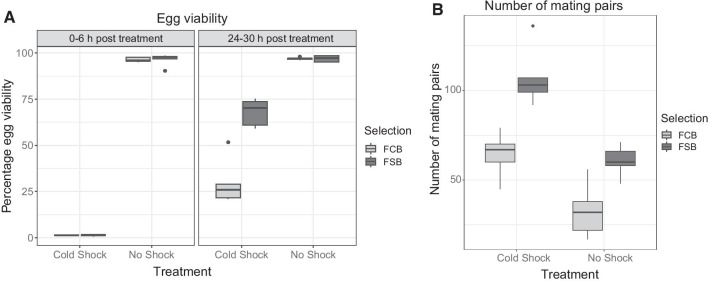
Mean mating frequency (number of mating pairs) and egg viability (Experiment 1). **A** We measured the egg viability at two different time-points post cold shock. Under the cold shock condition, both FSB and FCB populations had extremely low egg viability (~ 1.3%) of 0–6 h of measurement compared to 24–30 h of measurement or with no shock of 0–6 h and 24–30 h period of egg viability measurement. However, there was no significant difference in the egg viability measured 0–6 h for cold shock or no shock treatment between the FSB and FCB populations. Post cold shock, 24–30 h of egg viability had improved about 30–68% compared to egg viability of 0–6 h. Post cold shock, 24–30 h of egg viability was significantly different between FSB and FCB, FSB populations had higher egg viability ~ 2.27 times higher than FCB populations suggesting that the FSB population recovered faster than FCB population post cold shock. **B** Selections had significant effects on the number of mating pairs. Post cold shock, FSB populations had a roughly double number of mating pairs compared to the FCB populations. Treatment had significant effects on the mean number of mating pairs compared to no shock treatment, indicating that cold shock treatment had a significantly higher number of mating pairs than no shock treatment. The light gray box plot represents the FCB, and the dark gray box plot represents the FSB populations

Mean egg viability analysis reveals a significant effect of selection, period, treatment and three-way interaction selection × period × treatment (Table 1A). Egg viability of the FSB or FCB populations under no shock treatment was higher compared to egg viability of the FSB or FCB populations under cold shock treatment, suggesting that cold shock affects egg viability. Post cold shock, 0–6 h period had lower egg viability compared to 24–30 h period of egg viability (Fig. 1A). However, there was no significant difference in the egg viability of 0–6 h with and without cold shock between the FSB and FCB populations. Post cold shock, 24–30 h period of egg viability had improved about 30–68% compared to egg viability of 0–6 h. However, 24–30 h of egg viability was significantly higher (~ 2.27 times) in the FSB populations compared to FCB populations (Fig. 1A), suggesting that the FSB populations recovered faster than FCB populations post cold shock.

**Table 1:** Egg viability (Experiment 1) and mating frequency (Experiment 1)

Effect	SS	MS Num	DF Num	DF Den	*F* ratio	*p*
A. Egg viability (Experiment 1)						
Selection (Sel)	897.063	897.063	1.000	4.000	42.823	**0.003**
Period (Per)	5777.870	5777.870	1.000	4.000	160.943	**˂ 0.001**
Block (Blk)	197.531	49.383	4.000	1.024	1.282	0.570
Treatment (Trt)	51,295.911	51,295.911	1.000	4.000	1684.321	**˂ 0.001**
Sel × Per	904.019	904.019	1.000	4.000	94.232	**0.001**
Sel × Blk	83.793	20.948	4.000	0.010	14.312	0.964
Sel × Trt	912.133	912.133	1.000	4.000	58.506	**0.002**
Per × Blk	143.601	35.900	4.000	1.523	1.081	0.561
Per × Trt	5510.982	5510.982	1.000	4.000	116.449	**˂ 0.001**
Blk × Trt	121.820	30.455	4.000	2.018	0.777	0.629
Sel × Per × Blk	38.374	9.594	4.000	4.000	0.404	0.799
Sel × Per × Trt	904.254	904.254	1.000	4.000	38.122	**0.003**
Sel × Blk × Trt	62.362	15.590	4.000	4.000	0.657	0.653
Per × Blk × Trt	189.301	47.325	4.000	4.000	1.995	0.260
Sel × Per × Blk × Trt	94.881	23.720	4.000			
B. Mating frequency (Experiment 1)						
Selection (Sel)	6265.800	6265.800	1.000	4.000	29.187	**0.006**
Block (Blk)	499.700	124.925	4.000	0.950	0.701	0.705
Treatment (Trt)	7605.000	7605.000	1.000	4.000	39.947	**0.003**
Sel × Blk	858.700	214.675	4.000	4.000	0.946	0.521
Sel × Trt	304.200	304.200	1.000	4.000	1.341	0.311
Blk × Trt	761.500	190.375	4.000	4.000	0.839	0.565
Sel × Blk × Trt	907.300	226.825	4.000			

A: summary of results from a four-factor mixed model ANOVA on the egg viability using selection (FCB and FSB), treatment (cold shock and no shock), and period (0–6 h and 24–30 h) as fixed factors crossed with the random block (1–5). B: summary of results from a three-factor mixed model ANOVA on the number of mating pairs (mating frequency) using selection (FCB and FSB), and treatment (cold shock and no shock) as fixed factors crossed with the random block (1–5)

*p*-values in bold are statistically significant

The mating frequency showed response to cold shock. Selections had significant effects on the mating frequency (Table 1B). Post cold shock, FSB populations had a roughly double number of mating pairs compared to the FCB populations (Fig. 1B). Treatment had significant effects on the mean mating frequency. Compared to no shock treatment, cold shock treated populations had a significantly higher number of mating pairs. However, there was no significant effect of selection × treatment interaction on mating frequency (Table 1B). Increased egg viability in the FSB populations may be due to the more mating in FSB populations compared to FCB populations. The mating frequency and egg viability results align with the previous report [14].

#### Experiment 2.1: longevity assay

**Fig. 2:**
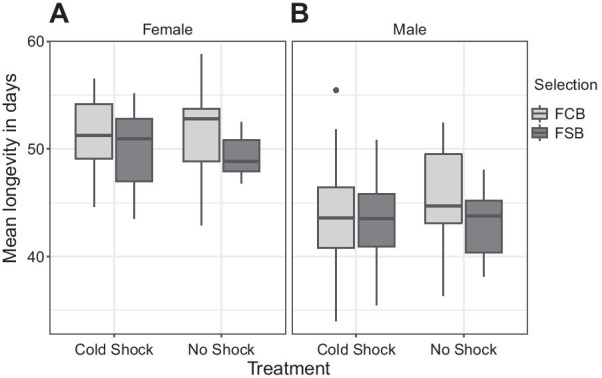
Longevity of males and females (Experiment 2.1). **A** Mean longevity of the FSB and FCB females after being exposed to cold shock or no shock treatment. Selection, treatment, or selection × treatment interaction did not have any significant effect on females’ mean longevity. **B** Mean longevity of the FSB and FCB males after being subjected to cold shock or no shock treatment. Selection, treatment, or selection × treatment interaction did not significantly affect mean males’ longevity. The light gray box plot represents the FCB, and the dark gray box plot represents the FSB populations

We found that the selected populations evolved cold shock resistance in the context of (a) egg viability 24–30 h post cold shock and (b) mating frequency over the first 36 h post cold shock. Hence, we assayed longevity to understand the costs associated with evolution of mating frequency and egg viability. We performed the longevity assay after 24 generations of selection. Male and female longevity were assessed in terms of mean, median, and maximum. Analyses of the mean longevity revealed that there was no significant effect of selection, treatment or selection × treatment interaction on female or male mean longevity (Table 2A, B; Fig. 2A, B). So the absence of significant effects of treatment and selection together suggest that the cold shock had no direct effects on the mean longevity (Table 2A, B). We also analyzed longevity data using the different parameters such as maximum longevity (Additional file 1: Table S1A, B) and median longevity of females and males (Additional file 1: Table S2A, B). However, just like in the case of mean longevity, we did not find a significant effect of the selection, treatment, and selection × treatment interaction on the maximum longevity, and median longevity.

**Table 2:** The mean longevity of males and females (Experiment 2.1)

Trait	Effect	SS	MS Num	DF Num	DF Den	*F* ratio	*P*
(A) Female longevity	Selection (Sel)	56.044	56.044	1.000	4.007	2.756	0.172
	Treatment (Trt)	1.581	1.581	1.000	4.004	0.047	0.839
	Block (Blk)	91.967	22.992	4.000	7.012	0.443	0.775
	Sel × Trt	0.160	0.160	1.000	4.071	0.084	0.786
	Sel × Blk	81.399	20.350	4.000	4.000	10.751	**0.020**
	Trt × Blk	133.984	33.496	4.000	4.000	17.697	**0.008**
	Sel × Trt × Blk	7.571	1.893	4.000	39.000	0.192	0.941
(B) Male longevity	Selection (Sel)	33.439	33.439	1.000	4.006	1.348	0.310
	Treatment (Trt)	14.549	14.549	1.000	4.008	0.752	0.435
	Block (Blk)	478.234	119.559	4.000	6.177	3.024	0.107
	Sel × Trt	9.169	9.169	1.000	4.032	1.972	0.232
	Sel × Blk	99.283	24.821	4.000	4.000	5.350	0.067
	Trt × Blk	77.398	19.349	4.000	4.000	4.170	0.098
	Sel × Trt × Blk	18.559	4.640	4.000	39.000	0.422	0.792

A: summary of results from a three-factor mixed model ANOVA on the females mean longevity using selection (FCB and FSB) and treatment (cold shock and no shock) as fixed factors crossed with the random block (1–5). B: summary of results from a three-factor mixed model ANOVA on the males mean longevity using selection (FCB and FSB) and treatment (cold shock and no shock) as fixed factors crossed with the random block (1–5)

*p*-values in bold are statistically significant

We found a significant effect of treatment on the *Gompertz a* (age-independent mortality rate) and b (age-dependent mortality rate) parameters among males. The FSB and FCB males subjected to cold shock showed significantly higher age-independent mortality but a significantly lower age-dependent mortality compared to the males not subjected to cold shock (Additional file 1: Table S3C, D). The net effect of these two factors was that the average (and median) lifespan of the males subjected to cold shock and those not subjected to cold shock was not different. There was no effect of selection or a selection × treatment interaction on the Gompertz parameters. Among the females, none of the factors affected the Gompertz parameters (Additional file 1: Table S3A, B). Thus, we found no evidence for any significant change in mean longevity or rates of aging as a correlated response to selection for increased resistance to cold shock.

#### Experiment 2.2: lifetime fecundity

**Fig. 3:**
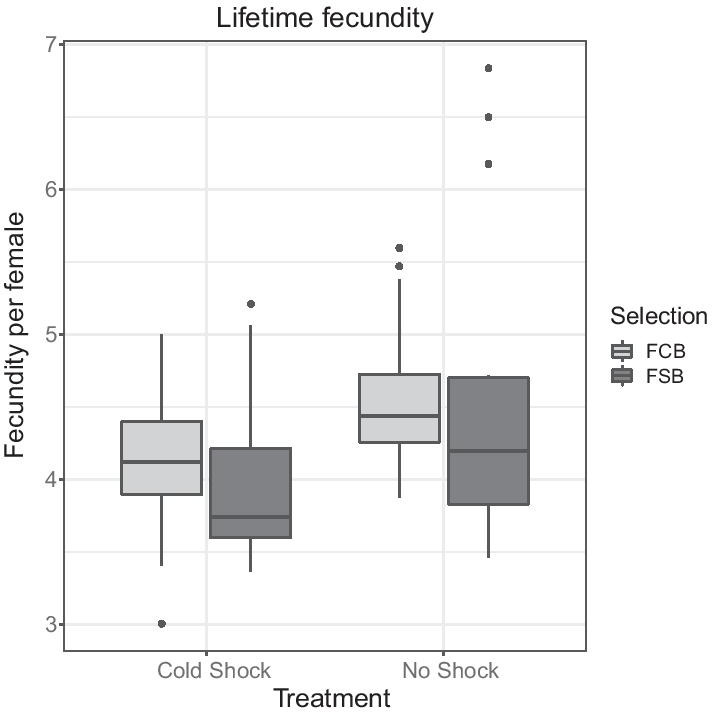
Mean lifetime fecundity per female (Experiment 2.2). Fecundity was measured at eleven-time points with age once in every 6 days, and a mean of eleven-time points of fecundity with age was computed. Selection, treatment or selection × treatment interaction did not have a significant effect on fecundity. Open bars represent the FSB populations, and closed bars represent the FCB populations. The light gray box plot represents the FCB, and the dark gray box plot represents the FSB populations

We measured another life-history trait, lifetime fecundity, to assess the cost associated with evolution of cold shock resistance. The mean number of eggs laid per female in each of the FSB and FCB populations with and without cold shock treatments was computed by averaging the eleven time points of fecundity measured with longevity assay and used it as the unit of analysis. We noticed treatment had a significant effect on lifetime fecundity, indicating that under cold shock condition both FSB and FCB populations had lower fecundity compared to no shock condition. However, we did not find the significant effect of selection, selection × treatment interaction on female fecundity (Table 3; Fig. 3). We also analyzed lifetime fecundity data using time-point (measure of fecundity with age) as a factor with repeated measures of ANOVA (Additional file 1: Table S4). We found that age had a significant effect on lifetime fecundity (Additional file 1: Table S4; Figure S1) indicating that fecundity reduces with age.

**Table 3:** Lifetime fecundity (Experiment 2.2)

Effect	SS	MS Num	DF Num	DF Den	*F* ratio	*P*
Selection (Sel)	0.006	0.006	1	4.001	0.004	0.955
Treatment (Trt)	4.264	4.264	1	4.009	24.427	**0.007**
Block (Blk)	13.135	3.284	4	1.650	2.639	0.328
Sel × Trt	0.017	0.017	1	4.002	0.023	0.886
Sel × Blk	7.161	1.790	4	4	2.485	0.199
Trt × Blk	0.698	0.174	4	4	0.242	0.901
Sel × Trt × Blk	2.881	0.720	4	39	5.699	**0.001**

Summary of results from a three-factor mixed model ANOVA on the lifetime fecundity using selection (FCB and FSB) and treatment (cold shock and no shock) as fixed factors crossed with the random block (1–5)

*p*-values in bold are statistically significant

#### Experiment 2.3: adult mortality

To probe the immediate effect of cold shock on adult mortality, 48 h post cold shock or no shock, we assessed the mortality of males and females along with longevity assay. We had chosen this time-point because this is time-point when we collect eggs to start next generation. Mean mortality analysis revealed that selection or sex had no significant effect on adult mortality (Additional file 1: Table S5). Treatment had a significant effect on adult mortality, suggesting that cold shock treatment significantly caused more adult mortality (~ 4%) (Additional file 1: Figure S2) than no shock treatment. Post cold shock, higher percentage of females died compared to males although this difference was not significant. Additionally, we did not find three-way interaction (selection × treatment × sex) significant (Additional file 1: Table S5). Our results suggest that the cold shock treatment that we used had a mild effect on the adult mortality. Hence, in our selection regime, the focus of selection was on egg viability.

#### Experiment 3.1: development time (first instar larva to adult eclosion)

**Fig. 4:**
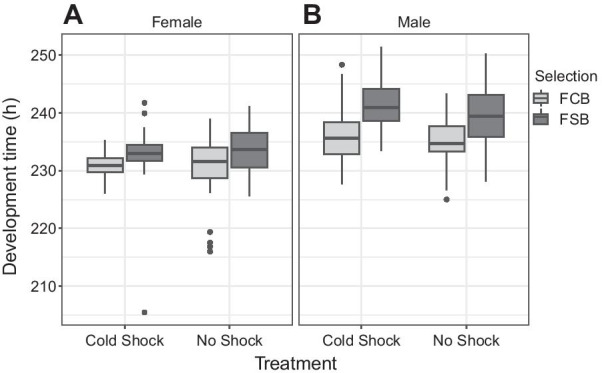
Development time of male and female (Experiment 3.1). **A** Development time (larva to adult) of the FSB and FCB females when their parents were subjected to cold shock or no shock treatments. We found a significant effect of the selection with FSB females developing ~ 2–6 h slower than FCB females. Treatment had no significant effect. **B** Development time (larvae to adults) of the FSB and FCB males when their parents were subjected cold shock or no shock treatments. We found a significant effect of selection regime with the FSB males developing 3–5 h slower than FCB males. Treatment had no significant effect. The light gray box plot represents the FCB, and the dark gray box plot represents the FSB populations

Unlike longevity and fecundity, selection did affect mean development time. Mean development time of males and females showed a significant effect of selection (Table 4A, B; Fig. 4A, B). Starting as first instar larvae, FSB males took about 3–5 h more time to emerge as adults than FCB males (Fig. 4B). Just like the males, FSB females also took ~ 2–6 h more time to emerge as adults compared to FCB females (Fig. 4A). However, the cold shock experienced by the parents had no effect on offspring development time (no significant effect of treatment). None of the other effects were significant.

**Table 4:** Developmental time (Experiment 3.1)

Trait	Effect	SS	MS Num	DF Num	DF Den	*F* ratio	*P*
(A) Female development time	Selection (Sel)	907.888	907.888	1.000	4.000	8.374	**0.044**
	Treatment (Trt)	206.835	206.835	1.000	4.000	0.453	0.538
	Block (Blk)	950.196	237.549	4.000	1.019	0.855	0.658
	Sel × Trt	204.729	204.729	1.000	4.000	0.712	0.446
	Sel × Blk	433.683	108.421	4.000	4.000	0.377	0.816
	Trt × Blk	1828.026	457.007	4.000	4.000	1.590	0.332
	Sel × Trt × Blk	1149.930	287.483	4.000	180.000	1.840	0.123
(B) Male development time	Selection (Sel)	758.240	758.240	1.000	4.000	17.449	**0.014**
	Treatment (Trt)	214.335	214.335	1.000	4.000	0.601	0.482
	Block (Blk)	141.786	35.446	4.000	3.993	0.098	0.977
	Sel × Trt	54.776	54.776	1.000	4.000	1.404	0.302
	Sel × Blk	173.816	43.454	4.000	4.000	1.114	0.460
	Trt × Blk	1427.513	356.878	4.000	4.000	9.150	**0.027**
	Sel × Trt × Blk	156.012	39.003	4.000	180.000	1.111	0.353

A: summary of results from a three-factor mixed model ANOVA on mean larvae to the adults development time of females using selection (FCB and FSB) and treatment (cold shock and no shock) as fixed factors crossed with the random block (1–5). B: summary of results from a three-factor mixed model ANOVA on the mean larvae to the adults development time of males using selection (FCB and FSB) and treatment (cold shock and no shock) as fixed factors crossed with the random block (1–5)

*p*-values in bold are statistically significant

#### Experiment 3.2: dry body weight of adults

**Fig. 5:**
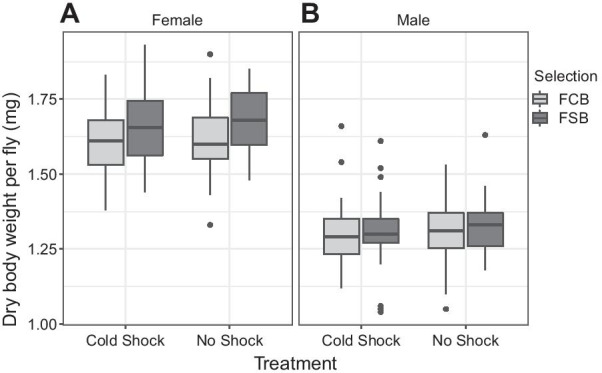
Dry weight at eclosion of males and females (Experiment 3.2). **A** The selection had a significant effect on females’ mean dry body weight. However, treatment or selection × treatment did not have significant effects on females’ mean dry body weight. Open bars represent the FSB populations, and closed bars represent the FCB populations. **B** Dry weight at eclosion of males from the FSB and FCB populations. Selection, treatment, or selection × treatment interaction did not significantly affect mean dry body weight. The light gray box plot represents the FCB, and the dark gray box plot represents the FSB populations

We found a significant effect of selection on females’ dry body weight, as FSB females were ~ 0.01 mg heavier than FCB females (Table 5A; Fig. 5A). However, there was no significant effect of treatment or selection × treatment interaction (Table 5A). Males’ mean dry body weight analysis revealed that there was no significant effect of selection, treatment, or selection × treatment interaction (Table 5B; Fig. 5B).

**Table 5:** Dry body weight of adult (Experiment 3.2)

Trait	Effect	SS	MS Num	DF Num	DF Den	*F* ratio	*P*
(A) Female dry body weight	Selection (Sel)	6.7 × 10^–3^	6.7 × 10^–3^	1	4	32.942	**0.005**
	Treatment (Trt)	3 × 10^–4^	3 × 10^–4^	1	4	0.287	0.621
	Block (Blk)	1.4 × 10^–2^	3.4 × 10^–3^	4	3.756	3.62	0.128
	Sel × Trt	2 × 10^–4^	2 × 10^–4^	1	4	1.199	0.335
	Sel × Blk	8 × 10^–4^	2 × 10^–4^	4	4	1.059	0.479
	Trt × Blk	3.7 × 10^–3^	9 × 10^–4^	4	4	4.828	0.078
	Sel × Trt × Blk	8 × 10^–4^	2 × 10^–5^	4	180	0.491	0.743
(B) Male dry body weight	Selection (Sel)	8.2 × 10^–5^	8.2 × 10^–5^	1	4	0.178	0.694
	Treatment (Trt)	7 × 10^–4^	7 × 10^–4^	1	4	0.701	0.45
	Block (Blk)	6 × 10^–3^	1.5 × 10^–3^	4	6.553	1.05	0.45
	Sel × Trt	1.2 × 10^–5^	1.2 × 10^–5^	1	4	0.24	0.65
	Sel × Blk	1.8 × 10^–3^	4.6 × 10^–4^	4	4	9.55	**0.025**
	Trt × Blk	4 × 10^–3^	1 × 10^–3^	4	4	20.974	**0.006**
	Sel × Trt × Blk	2 × 10^–4^	4.8 × 10^–5^	4	180	0.145	0.965

A: summary of results from a three-factor mixed model ANOVA on the mean dry body weight of females using selection (FCB and FSB) and treatment (cold shock and no shock) as fixed factors crossed with random block (1–5). B: summary of results from a three-factor mixed model ANOVA on the mean dry body weight of males using selection (FCB and FSB) and treatment (cold shock and no shock) as fixed factors crossed with the random block (1–5)

*p*-values in bold are statistically significant

#### Experiment 3.3: larvae to adults survival

**Fig. 6:**
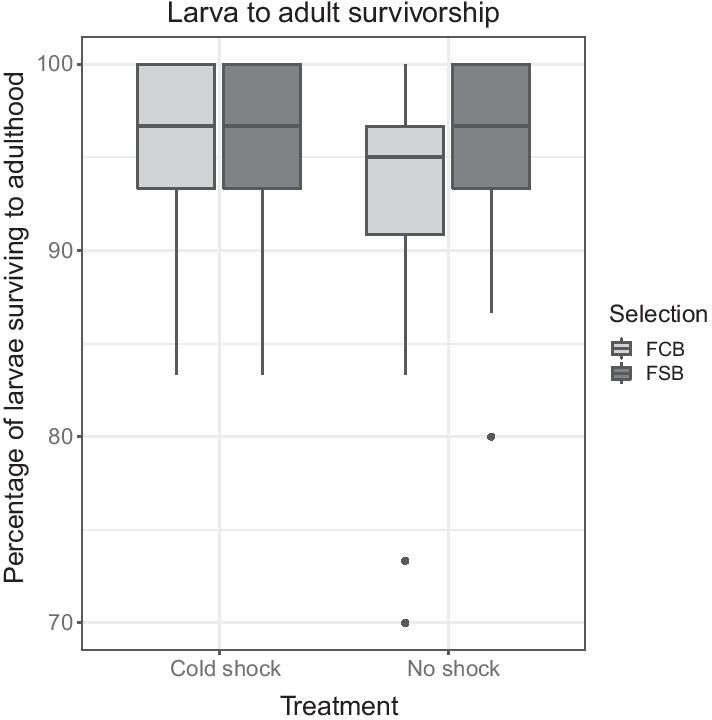
Larvae to adults survival (Experiment 3.3). Selection, treatment, or selection × treatment did not significantly affect mean larvae to adults survival. The light gray box plot represents the FCB, and the dark gray box plot represents the FSB populations

Mean larvae to adults survival analysis showed no significant effect of selection, treatment, or selection × treatment interaction (Table 6; Fig. 6). This indicates that cold shock treatment does not affect the survival of larva to adulthood.

**Table 6:** Larvae to adults survival (Experiment 3.3)

Effect	SS	MS Num	DF Num	DF Den	F ratio	Prob > F
Selection (Sel)	37.556	37.556	1	4	2.449	0.193
Treatment (Trt)	128.000	128.000	1	4	3.578	0.132
Block (Blk)	1270.889	317.722	4	1.918	10.417	0.096
Sel × Trt	107.556	107.556	1	4	5.218	0.084
Sel × Blk	61.333	15.333	4	4	0.744	0.609
Trt × Blk	143.111	35.778	4	4	1.736	0.303
Sel × Trt × Blk	82.444	20.611	4	180	1.013	0.402

Summary of results from a three-factor mixed model ANOVA on the mean larvae to adults survival considering selection (FCB and FSB) and treatment (cold shock and no shock) as fixed factors crossed with the random block (1–5)

## Discussion

The evolution of higher egg viability and mating frequency in response to cold shock resistance, observed in our current and previous study [14], may be costly. Therefore, it is possible that allocation of resources to these traits can lead to trade-offs with other important life-history traits. So far, none of the known studies have explored the life-history cost associated with evolution of mating frequency and egg viability in response to cold shock resistance. Therefore, we assessed mating frequency, egg viability, mean longevity, rates of aging, fecundity, development time, dry body weight, and larvae to adults survival in the cold shock selected populations (FSB) and their control populations (FCB). Neither longevity nor fecundity was different between the FSB and FCB populations. However, we found that males and females from the FSB populations took significantly more time to develop (from first instar larva to adult) relative to the FCB populations. Females from the FSB populations were heavier than females from the FCB populations. However, there was no difference in male body size between the FSB and FCB populations. Additionally, we did not notice the significant difference in larvae to adults survival between FSB and FCB populations. Taken together, our finding suggests there is no evidence for a trade-off between the ability to resist cold stress and important life-history traits.

The correlation between cold shock resistance and longevity is variable across studies. MacMillan et al. [24], using a selection protocol very similar to the present study found that females of the cold shock selected populations had decreased longevity compared to females of the control populations whereas no such difference was visible in the males. According to Anderson et al. [35] populations selected for faster chill-coma recovery had reduced lifespan compared to controls. On the contrary, Norry and Loeschcke [36] observed that cold-adapted populations lived longer at 14 °C and shorter at 25 °C compared to control populations. Bubliy and Loeschcke [7] found no change in female longevity between populations selected for cold resistance and their controls. In populations directly selected for increased lifespan, increased cold resistance evolved as a correlated response in adults and pupae of *D. melanogaster* [37]. Similar to Bubbly and Loeschcke studies [7], we also found that selection for increased resistance to cold shock had no effect on lifespan or rates of aging. However, our results are different from other studies due to several possible differences, including the base population used for selection, the definition of ‘cold stress’, the assay protocols, etc., between these studies that preclude a direct comparison of results. More importantly, other studies typically selected for increased survivorship post cold shock. However, in our study cold shock induced very low levels of adult mortality (about 1.5–5%), while it drastically reduced egg viability (~ 30–68%). This is further strengthened by the fact that the lifespan of the FSB and FCB populations that were subjected to cold shock were not different from the longevity of those populations not subjected to cold shock. Thus, it is not surprising that longevity did not evolve in the FSB compared to FCB populations.

In several previous studies, fecundity has responded to selection for cold resistance. Anderson et al. [35] found the higher fecundity in their selection regime under cold shock condition. Watson and Hoffmann [34] found that cold selected populations had lower fecundity. However, we found no difference in the lifetime fecundity between FSB and FCB populations. This result is in agreement with our earlier, short-term measurement of fecundity in these two populations [14]. Thus, we found no evidence of a trade-off between evolved cold stress resistance and fecundity.

Increased development time could be a cost in species like *D*. *melanogaster* that inhabit ephemeral habitats and have to complete their development before the habitat disappears. We did find that the FSB males and females had increased developmental time. However, the magnitude of the increased developmental time of the FSB males (~ 3–5 h) and females ~ 2–6 h) was minimal, and hence we are not sure whether this could represent a cost. During the late third instar larval stage, *D. melanogaster* larvae feed rapidly and increased their weight exponentially [38]. An increase feeding time of males (~ 3–5 h) and females (~ 2–6 h) can increase the number of resources stored by the larvae during this period. Accordingly, populations of *D. melanogaster* selected for increased starvation and desiccation stress resistance show increased development time and increased body size [39, 40]. In this study, increased development time represented an adaptation to acquire necessary resources to cope with cold stress.

Body weight at eclosion is often used as a proxy for the number of resources stored by the larvae. Anderson et al. [35] and Watson and Hoffmann [34] found no difference in the body size of flies selected for increased cold resistance. In this study, FSB females were heavier at eclosion compared to FCB females. This indicated that FSB females were storing extra/specific nutrients to survive cold shock. However, there was no difference in body weight between FSB and FCB males. Taken together, this indicated that, at least in females, increased development time was likely to be beneficial in the aspect of increased resource acquisition. It is also to be noted that in our previous study, females suffered more mortality post cold shock relative to males [14].

The absence of any change in lifespan and fecundity of the FSB populations could be because of many reasons. Firstly, the evolved cold shock resistance of the FSB populations might be very cheap. Thus, the resources required to combat the effects of cold stress in our selection regime might be very low. A second alternative is the food used in our selection regime was indeed rich, and the larval and adult densities of FSB populations were low. Therefore, it was possible that our flies inhabited resource-rich environment. If this is true, then assays under resource-depleted condition should lead to different results. Finally, it is quite possible that the cost of increased cold resistance is paid in a different currency. While we did not find any difference in adult longevity or fecundity, other traits that we have not measured here might have been reduced in the FSB populations. The possible set of such traits include starvation and desiccation resistance.

## Conclusions

Our findings revealed that there are no apparent life-history trade-offs between increased resistance to cold shock (in the aspect of increased reproductive traits and egg viability post cold shock) with life-history traits i.e. the longevity, lifetime fecundity, larvae to adults survival, adult mortality, and larva to adult developmental time, which indicated that evolved cold stress resistance need not come at the cost of life-history traits. However, it is possible that the cost of increased cold stress resistance is paid in terms of reduced resistance to other stresses.

## Methods

### Experimental populations

Details of the maintenance and derivation of the selected (FSB; Cold Shock Selected populations derived from Blue Ridge Base (BRB) line populations) and their control (FCB; Cold Shock Control populations derived from BRB population) populations have been explained in the previous reports [14]. Briefly, in 2010, we created the BRB population by mixing 100 individuals of male and females from each of the 19 isofemale lines. Original 19 isofemale lines were kindly gifted to us from Prof. Daniel Promislow Laboratory. These isofemale lines were originally established in Prof. Daniel Promislow’s Laboratory from the inseminated wild females of *D. melanogaster* that were collected from Blue Ridge, Georgia, USA. After receiving these lines, from Promislow’s Laboratory we further maintained them for six generations at the standard laboratory conditions. After that, we combined 100 males and females from each of the 19 isofemale lines to create a large population and named the populations as BRB. We maintained the BRB population in the laboratory for 10 generations. After that, we split the BRB population into 5 replicate populations referred to as “BRB 1–5”. We maintained BRB 1–5 populations for 35 generations at standard laboratory conditions (more details see the flow chart, Additional file 1: Figure S3).

After 35 generations of the laboratory adaptation of BRB 1–5, an FSB and an FCB population were established from each of the five BRB replicate populations, for example, FSB 1 and their corresponding control FCB 1 originated from the BRB 1, similarly FSB 2 and its control FCB 2 created from the BRB 2, and so on. Hence, we had five replicate populations for FSB, and FCB carrying the same numeral have originated from the same baseline population (BRB) and are closer to each other than any other population. For instance, FSB 1 and FCB 1 are closer to each other due to the origin from the same ancestral population than FSB 2 or FCB 2 or any other population. Hence, in our statistical data analysis, FSB 1 and FCB 1 are included in block 1; similarly, FSB 2 and FCB 2 are included in block 2, and so on.

### Cold shock selection protocol

The FSB and FCB populations are large outbred populations maintained under the standard laboratory environment (25 °C temperature, 50–60% relative humidity, 12 h light: 12 h dark cycles, on a 13-day discrete generation cycle). On day 12 post egg collection, flies (roughly 2–3 days old as adults and mated) are moved into empty, clean, dry glass vials (30 mm diameter × 90 mm length). After that, flies belonging to the FSB populations are subjected to − 5 °C temperature in an ice-salt-water slurry for 1 h. FCB populations, on the other hand, are held at 25 °C for 1 h. Subsequently, all populations are quickly moved into a separate Plexiglass cage (25 cm length × 20 cm width × 15 cm height) having fresh banana–yeast–jaggery food (hereafter referred to as “food”) plate. After 24 h, a fresh food plate is given to flies to oviposit for 18 h to collect eggs to initiate the next generation. For FCB populations, 20 vials are collected at a density of ~ 70 eggs per vial containing ~ 6 mL of fresh food and for FSB populations 20 vials are collected at a density of ~ 100 egg/vial. We collected different density of eggs for FSB and FCB populations. Because of egg hatchability differences, the number of larvae were about 70 per vial in each population. Therefore, the number of adults were about 1200–1400 per population in both FCB and FSB. Experiements were perofmed in this study over 24–33 generations of selection. In our selection regime, we used non-lethal temperature − 5 °C for 1 h, which induces only ~ 1.5–5% adult mortality. However, our selection regime is acting on egg viability because at 0–6 h or 24–30 h post cold shock egg viability is drastically reduced egg to ~ 98%, and ~ 75%, respectively. Therefore, this results suggest that our selection regime is presumably acting on egg viability instead of adult mortality.

### Standardization

To account for the non-genetic parental effects [41], flies from the selected populations and their controls were reared for one generation in a common rearing environment. This method is referred to as standardization, and these flies are known as standardized flies. A detail of the standardization protocol has been described earlier in Singh et al. [14]. Shortly, to control egg density, 20 vials were established at a density of 70 eggs per vials in ~ 6 mL food for each selected and their control populations, reared at standard laboratory conditions (12 h light:12 h dark). On day 12, after egg collection (roughly 2–3 days old as adult flies), ~ 1400 flies of each population were transferred separately in a Plexiglass cage and provided a fresh food plate. These flies were further used for experiment egg collection.

### Cold shock treatment for experiments

A detailed account of the cold shock protocol has been described in our previous study [14]. In short, on day 12 post egg collection (by this time, flies were roughly 2–3 days old as an adult and mated flies), 25 pairs of males and females were moved to clean, dry glass vials under mild carbon dioxide anesthesia. The cotton plug was inserted deep into the vial such that the flies were allowed to stay in a confined space in the vial (1/3 of the vial). The flies were kept in an incubator to recover from carbon dioxide anesthesia for half an hour. The vials containing flies were then kept for 1 h in an ice-salt-water slurry maintained at − 5 °C. Post cold shock, flies were quickly shifted to Plexiglass cages (14 cm length × 16 cm width × 13 cm height. The cage was provided with a food plate and was kept under standard laboratory conditions [14]. The control treatment flies were handled similarly, except that the vials containing flies were kept in a water bath maintained at 25 °C for 1 h.

## Experimental details

### Experiment 1: mating frequency and egg viability

To investigate cold shock resistance response in context of egg viability and mating frequency post cold shock or no shock conditions, we repeated the experiment on 24 generations post selection similar to the previously reported [14] to understand whether the previously observed response persistent across several generations post cold shock selection so that we can investigate the cost associated with evolution of mating frequency, and egg viability. We set up ten vials at a density of 70 eggs/vial from each of the FSB 1–5 and FCB 1–5 populations. On day 12 post egg collections, four vials of 25 pairs of males and females were collected using mild carbon dioxide anesthesia from each of the FSB 1–5, FCB 1–5 populations for cold shock, or no shock treatments. Cold shock or no shock treatment was subjected to these flies using the above-mentioned cold shock protocol. Soon after cold shock flies were transferred to the Plexiglas cage at a density of 100 pairs of male and female per cage and provided a fresh food plate to estimate the egg viability for 0–6 h period and 24 h later another fresh food plate was given to measure the egg viability for 24–30 h period. Mating pairs were observed from the same cage at every 30 min intervals for 0–36 h post cold shock. We had chosen the time point 0–6 h and 24–30 h period for egg viability because 0–6 h period represents the immediate effect of cold shock on the egg viability. However, 24–30 h represents the time of normal selection regime where we collect eggs to start the next generation for both the FSB 1–5 and FCB 1–5 populations (see the details of the experimental design in the illustration, Additional file 1: Figure S4).

### Experiment 2.1: longevity assay

To assess the cost associated with evolution of mating frequency and egg viability, we performed longevity assay after 24 generations of selection. Eggs were collected from standardized flies at a controlled egg density of 70 eggs/vial provisioned with ~ 6 mL of fresh food. Twenty-four such vials were set up for each of the FSB 1–5 and FCB 1–5 populations. On day 12 after egg collection, flies were sorted (25 mating pairs per vial) under mild carbon dioxide anesthesia. After sorting, flies were divided into two sets: (a) set first for cold shock treatment (both male and female flies were exposed to cold shock for 1 h) (b) set second for no shock treatment (neither males and nor females were exposed to cold shock).

- Cold shock: For each population, flies contained in 12 vials (each vial contains 25 mating pairs of male and female) were imposed cold shock (− 5 °C for 1 h) as mentioned in the cold shock protocol. Quickly, after the cold shock, 12 vials were randomly divided into 3 sets referred to as a “replicate”. Each set with 4 vials of flies (100 mating pairs each) was moved into a Plexiglass cage and given a fresh food plate. Hence, each population (FSB 1–5 and FCB 1–5) had 3 replicates.
- No shock: For each population, flies contained in 12 vials (each vial contain 25 mating pairs of male and female) were subjected to no shock treatment (25 °C for 1 h). Soon after the treatment, 12 vials were randomly divided into three sets of 4 vials that were known as replicate. Each set having 4 vials containing total of 100 mating pairs of males and females flies were moved into Plexiglas cages and given a fresh food plate. Hence, each of the population FSB 1–5 and FCB 1–5 had 3 replicates.

We established three replicate cages per selection × block × treatment combination (except block 1 of the FCB population, which had two replicates for both cold shock or no shock treatment, due to accidental death of one of the replicates during the assay). The food plate was changed 48 h internal and dead flies were aspirated out and computed. The sex of the dead flies was determined under the microscope based on sex combs. Mortality was recorded until the last fly died. Using the mortality data for each cage, we measured the mean longevity, median longevity, and maximum longevity of males and females from the selection regime (FSB and FCB), treatment, and block (see the details of the experimental design in illustration, Additional file 1: Figure S5).

### Experiment 2.2: lifetime fecundity

We measured another life-history trait to assess the cost associated with the evolution of egg viability and mating frequency as response to cold shock resistance. Hence, we assayed the lifetime fecundity along with longevity assay, using the same set of flies. The fecundity was measured at every 6th day along with longevity, i.e. at 11 time points. In order to measure fecundity, a fresh food plate was placed in the Plexiglas cage for 6 h for oviposition. After that, total number of eggs on each plate was counted under the microscope. Subsequently, fecundity per female was calculated using the formula in the bracket (fecundity per female = total number of eggs of a time point/the total number of live females at that time point). To measure the lifetime fecundity, we computed the average fecundity of the eleven-time points that were measured with longevity was calculated for three replicates for each of the FSB 1–5 and FCB 1–5 populations, and treatments (cold shock vs. no shock), except for the FCB 1 populations with cold shock treatment which had only two replicates (see the details of experimental design in the illustration, Additional file 1: Figure S5).

### Experiment 2.3: adult mortality

To investigate the effect of cold shock on adult mortality, 48 h post cold shock or no shock treatment, we assessed males and females mortality along with longevity assay. Forty-eight hour post cold shock, number of dead males and females were counted from each of the FSB 1–5 and FCB 1–5 for both cold shock or no shock treatment and percentage of adult mortality was computed and used as a unit of analysis (see the details for the experimental design in illustration, Additional file 1: Figure S5).

### Experiment 3.1: development time (first instar larva to eclosion)

Development time was assayed after 33 generations of selection. Followed by one generation of common rearing environment (no selection was imposed on FSB and FCB population), 12 vials each were set up for FSB 1–5 and FCB 1–5 populations at a density of 70 eggs per vial. On day 12, after egg collection, vials containing flies were randomly divided into two sets for -(a) cold shock, and (b) no shock treatment. After that, the flies were subjected to cold shock or no shock treatments, following the protocol as mentioned above. Immediately after cold shock treatment, flies (200 males and 200 females) were transferred to Plexiglass cage and provided with a fresh food plate. Twenty four hours post cold shock, fresh food plates were given to each cage for 1 h to lay stored eggs. After that, another set of fresh plates were given for 4 h. The second set of plates containing eggs were then incubated at standard laboratory conditions for 18 h to allow eggs to hatch and first instar larvae to emerge. The first instar larvae were collected (using a moist brush) into vials with 6 mL of fresh food. For each population and treatment combination, 10 replicate vials were set up (each containing 30 larvae in 6 mL of food). The vials were incubated at standard laboratory conditions. The positions of the vials were randomized and moved daily within the incubator. Once pupae formed, each vial was manually scanned every 2 h. Freshly eclosed flies were transferred into empty glass vials, sexed, and counted. The flies were then flash-frozen using liquid nitrogen and then transferred to − 80 °C for storage used to assess dry body weight (see the details of the experimental design in the illustration, Additional file 1: Figure S6). Mean larva to eclosion development time was computed for each vial, and these vials mean development time were used for the analysis.

### Experiment 3.2: dry body weight adult

To measure the dry body weight, we used the same flies from the development time assay (mentioned above). Freshly eclosed flies were flash-frozen using liquid nitrogen and stored at − 80 °C until dry body weight measurement. Five flies of given sex were grouped, dried in a hot air oven at 65 °C for 48 h, and weighed. For each population, treatment, and sex combination, ten such sets were weighed. Thus, a total of 50 males and 50 females per population and treatment were used for body weight measurement. Body weight of each group of five flies was considered as the unit of analysis (see the details for the design of the experiment in the illustration, Additional file 1: Figure S6).

### Experiment 3.3: larvae to adults survival

To investigate larvae to adults survival, we monitored the total number of flies eclosed from the cultured larval vial at a density of 30 larvae/vial from the development time experiment (see the details for the design of experiment in the illustration, Additional file 1: Figure S6). We calculated the percentage of larvae to adults survival using the equation given in a bracket (percentage of larvae to adults survival = (number of eclosed flies in a vial/total number of larvae cultured in a vial) × 100).

### Statistical analysis

Experiment 1: egg viability percentage was analysed using four-factor mixed model analysis of variance (ANOVA) considering selection regime (FSB vs. FCB), treatment (cold shock vs. no shock), period (0–6 h vs. 24–30 h) as a fixed factors crossed with a random block (1–5). The number of mating frequency was analyzed using three-factor mixed model ANOVA considering selection regime (FSB vs. FCB), treatment (cold shock vs. no shock) as a fixed factors crossed with a random block (1–5). Mean longevity, median longevity, maximum longevity (Experiment 2.1), larva to adult development time (Experiment 3.1), the dry body weight of males and females (Experiment 3.2), and larvae to adults survival (Experiment 3.3) data were analyzed using a three-factor mixed model ANOVA treating selection regime (FSB vs. FCB), treatment (cold shock vs. no shock) as a fixed factors crossed with a random block (1–5). The sexes were analyzed separately. Percentage adult mortality was analyzed using four-factor mixed model ANOVA treating selection regime (FSB vs. FCB), sex (male vs. female), and treatment (cold shock vs. no shock) as fixed factors crossed with a random block (1–5) (Experiment 2.3). Lifetime fecundity per female (Experiment 2.2) was analyzed using a three-factor mixed model ANOVA treating selection regime (FSB vs. FCB) and treatment (cold shock vs. no shock) as fixed factors crossed with block as a random factor. All the analyses were done at α = 0.05 level of significance using JMP Pro, Version 15, Statsoft. Multiple comparisons were carried out employing Tukey’s HSD.

To evaluate whether data are normally distributed we fit linear mixed-effects models (comparable to our analysis in JMP) using ‘lme4’ package in R and subsequently plotted the histograms of the residuals and tested the normality of the residuals using the Shapiro test. We noted the mean longevity, lifetime fecundity, and mating frequency data follows normality. However, data for the development time, dry body weight, and larvae to adults survival, egg viability p-values are less than < 0.05, though residuals plot seems normal for development time, dry body weight see Additional file 1. We also did non parametric Kruskal–Wallis test on the development time, dry body weight, egg viability (see below) and we found that the conclusion of the results remains the same. We have also added the results of Shapiro test of egg viability, mating frequency, longevity, lifetime fecundity, dry body weight, adult mortality, and larvae to adults survival in Additional file 1. Moreover, we added the Kruskal–Wallis test results that was conducted on the egg viability, development time, dry body weight, and adults mortality in the Additional file 1. Furthermore, we also analyzed data of egg viability, mean longevity, lifetime fecundity, adult mortality, development time, and larva to adult survivorship using the different method that is Generalized Linear Model (GLM) and we found that conclusion of results remains same.

#### Rates of aging

The age-dependent and age-independent rate of aging was measured using the method used by Mueller et al. [42, 43]. Raw survivorship data were used to calculate ‘proportion survival’ values with subsequent calculation of the running average of the proportion survival data, *r*_*x*_.

1 $$r_{x} = \left( {p_{x} + p_{x + 2} } \right)/2$$

where *p*_*x*_ is the proportion of individuals surviving at a given age *x*. Since mortality was monitored every other day, *x* and *x* + 2 are two successive age intervals noticed. The hazard rate that is the probability of death per unit time, *μ*_*x*_ at age x was computed employing the following equation:

2 $$\mu_{x} = {{\left( {r_{x} {-} \, r_{x + 2} } \right)} \mathord{\left/ {\vphantom {{\left( {r_{x} {-} \, r_{x + 2} } \right)} {r_{x} }}} \right. \kern-\nulldelimiterspace} {r_{x} }}$$

According to the Gompertz equation, the mortality rate at age *x* is given by,

3 $$\mu_{x} = ae^{bx}$$

where *a* and *b* represent age-independent and age-dependent rates of aging, respectively. Log-hazard rate was regressed against age intervals; the intercept and the least square slope gave the estimates of *Gompertz a* and *Gompertz b* respectively. The derived parameters were analyzed using three factors mixed model ANOVA with selection regime (FSB vs. FCB), treatment (cold shock vs. no shock) as fixed factor crossed with random blocks (1–5) (Experiment 2.1).

## Supplementary Information

**Additional file 1:** Results of the longevity data were analyzed using different parameters such as maximum longevity, median longevity, age-independent, and age-dependent longevity. The result of the lifetime fecundity data was analyzed using repeated measures of mixed-model ANOVA. Illustrations for the experimental design. Results of the effect of cold shock on adults mortality. Analysis of the data for normality test. Analysis of data using a non-parametric test, and generalized linear model (GLM).

## Data Availability

All data analyzed or used for this study are available from the DRYAD database [https://datadryad.org/stash/share/bQSA1KWPNLrY_pATfFZEfU3KFmzK5OVbqiz6SONul0A].

## Abbreviations

FSB: Freeze shock selected line derived from the BRB populations
FCB: Freeze shock control populations derived from the BRB populations
BRB: Blue Ridge Baseline populations
ANOVA: Analysis of variance
H: Hour
