# Supplementary material for: Evolution of reproductive traits have no apparent life-history associated cost in populations of Drosophila melanogaster selected for cold shock resistance
Source: BMC Ecol Evol. 2021 Dec 6;21:219. doi: 10.1186/s12862-021-01934-2 (PMC8650462; doi:10.1186/s12862-021-01934-2)
Supplement: Supplementary file 1 — Additional file 1: Results of the longevity data were analyzed using different parameters such as maximum longevity, median longevity, age-independent, and age-dependent longevity. The result of the lifetime fecundity data was analyzed using repeated measures of mixed-model ANOVA. Illustrations for the experimental design. Results of the effect of cold shock on adults mortality. Analysis of the data for normality test. Analysis of data using a non-parametric test, and generalized linear model (GLM). [file 12862_2021_1934_MOESM1_ESM.docx]

**Additional file 1**

**Supp. Table 1A and B.** Effect of cold shock on the maximum longevity of female and male (Experiment 2.1). Summary of results from a three-factor mixed model ANOVA on (A) female and (B) male using Selection (FCB and FSB) and Treatment (cold shock and no shock) as fixed factors crossed with the random block (1-5). *p*-values in bold are statistically significant.

| **Trait** | **Effect** | **SS** | **MS Num** | **DF Num** | **DF Den** | ***F* ratio** | ***p*** |
| --- | --- | --- | --- | --- | --- | --- | --- |
|  | Selection (Sel) | 42.2927 | 42.2927 | 1.0000 | 4.0177 | 2.3714 | 0.1981 |
| (A) | Treatment (Trt) | 30.0650 | 30.0650 | 1.0000 | 4.0287 | 2.7337 | 0.1731 |
| Female | Block (Blk) | 689.7273 | 172.4318 | 4.0000 | 0.0421 | . | . |
| Maximum | Sel × Trt | 7.1707 | 7.1707 | 1.0000 | 4.0096 | 0.2188 | 0.6643 |
| Longevity | Sel × Blk | 71.3030 | 17.8258 | 4.0000 | 4.0000 | 0.5435 | 0.7153 |
|  | Trt × Blk | 43.9091 | 10.9773 | 4.0000 | 4.0000 | 0.3347 | 0.8429 |
|  | Sel × Trt × Blk | 131.1818 | 32.7955 | 4.0000 | 39.0000 | 1.4201 | 0.2454 |
| (B) Male | Selection (Sel) | 21.0732 | 21.0732 | 1.0000 | 4.0033 | 0.1512 | 0.7172 |
| Maximum | Treatment (Trt) | 283.3171 | 283.3171 | 1.0000 | 4.0109 | 6.7764 | 0.0597 |
| Longevity | Block (Blk) | 28.2652 | 7.0663 | 4.0000 | 2.3818 | 0.0581 | 0.9898 |
|  | Sel × Trt | 21.0732 | 21.0732 | 1.0000 | 4.0076 | 0.3523 | 0.5847 |
|  | Sel × Blk | 558.3864 | 139.5966 | 4.0000 | 4.0000 | 2.3323 | 0.2161 |
|  | Trt × Blk | 167.2955 | 41.8239 | 4.0000 | 4.0000 | 0.6988 | 0.6316 |
|  | Sel × Trt × Blk | 239.4167 | 59.8542 | 4.0000 | 39.0000 | 1.7938 | 0.1497 |

**Supp. Table 2A and B.** Effect of cold shock on the median longevity of female and male (Experiment 2.1). Summary of results from a three-factor mixed model ANOVA on (A) female and (B) male using Selection (FCB and FSB) and Treatment (cold shock and no shock) as fixed factors crossed with the random block (1-5). *p*-values in bold are statistically significant.

| **Trait** | **Effect** | **SS** | **MS Num** | **DF Num** | **DF Den** | ***F* ratio** | ***p*** |
| --- | --- | --- | --- | --- | --- | --- | --- |
|  | Selection (Sel) | 54.699 | 54.699 | 1.000 | 4.005 | 1.501 | 0.288 |
| (A) Female | Treatment (Trt) | 84.293 | 84.293 | 1.000 | 4.012 | 5.499 | 0.079 |
| Median | Block (Blk) | 96.066 | 24.017 | 4.000 | 3.590 | 0.611 | 0.680 |
| Longevity | Sel × Trt | 1.041 | 1.041 | 1.000 | 4.015 | 0.083 | 0.787 |
|  | Sel × Blk | 145.930 | 36.482 | 4.000 | 4.000 | 2.919 | 0.162 |
|  | Trt × Blk | 61.324 | 15.331 | 4.000 | 4.000 | 1.227 | 0.424 |
|  | Sel × Trt × Blk | 49.991 | 12.498 | 4.000 | 39.000 | 0.940 | 0.451 |
| (B) Male | Selection (Sel) | 19.354 | 19.354 | 1.000 | 4.007 | 0.676 | 0.457 |
| Median | Treatment (Trt) | 1.175 | 1.175 | 1.000 | 4.008 | 0.046 | 0.841 |
| Longevity | Block (Blk) | 473.036 | 118.259 | 4.000 | 3.751 | 2.976 | 0.166 |
|  | Sel × Trt | 18.248 | 18.248 | 1.000 | 4.014 | 1.260 | 0.324 |
|  | Sel × Blk | 114.536 | 28.634 | 4.000 | 4.000 | 1.978 | 0.263 |
|  | Trt × Blk | 102.324 | 25.581 | 4.000 | 4.000 | 1.767 | 0.297 |
|  | Sel × Trt × Blk | 57.915 | 14.479 | 4.000 | 39.000 | 0.986 | 0.426 |

**Supp. Table 3A and B.** Effect of cold shock on the age-independent and age-dependent mortality rates of female (Experiment 2.1). Summary of results from a three-way mixed-model ANOVA on (A) age-independent and (B) age-dependent mortality rate among females using selection (FCB and FSB) and treatment (cold shock and no shock) as fixed factors crossed with the random block (1-5).

| **Trait** | **Effect** | **SS** | **MS Num** | **DF Num** | **DF Den** | ***F* ratio** | ***p*** |
| --- | --- | --- | --- | --- | --- | --- | --- |
| (A) Female | Selection (Sel) | 0.005 | 0.005 | 1.000 | 4.008 | 0.014 | 0.913 |
| age | Treatment (Trt) | 0.240 | 0.240 | 1.000 | 4.007 | 0.597 | 0.483 |
| independent | Block (Blk) | 0.600 | 0.150 | 4.000 | 1.491 | 0.366 | 0.822 |
| Mortality | Sel × Trt | 0.092 | 0.092 | 1.000 | 4.008 | 0.245 | 0.647 |
|  | Sel × Blk | 1.534 | 0.384 | 4.000 | 4. 000 | 1.019 | 0.493 |
|  | Trt × Blk | 1.611 | 0.403 | 4.000 | 4.000 | 1.07 | 0.475 |
|  | Sel × Trt × Blk | 1.505 | 0.376 | 4.000 | 39.000 | 1.74 | 0.161 |
| (B) Female | Selection (Sel) | 1.4×10^-4^ | 1.5×10^-4^ | 1.000 | 4.008 | 0.904 | 0.395 |
| age | Treatment (Trt) | 5.2×10^-4^ | 5.1×10^-4^ | 1.000 | 4.012 | 4.646 | 0.097 |
| dependent | Block (Blk) | 6.9×10^-4^ | 1.7×10^-4^ | 4.000 | 1.536 | 1.204 | 0.532 |
| Mortality | Sel × Trt | 1.3×10^-4^ | 1.3×10^-4^ | 1.000 | 4.010 | 1.038 | 0.366 |
|  | Sel × Blk | 6.4×10^-4^ | 1.6×10^-4^ | 4.000 | 4.000 | 1.265 | 0.413 |
|  | Trt × Blk | 4.4×10^-4^ | 1.1×10^-4^ | 4.000 | 4.000 | 0.871 | 0.552 |
|  | Sel × Trt × Blk | 5.1×10^-4^ | 1.3×10^-4^ | 4.000 | 39 .000 | 1.355 | 0.267 |

**Supp. Table 3C and D.** Effect of cold shock on the age-independent and age-dependent mortality rates of male (Experiment 2.1). Summary of results from a three-factor mixed model ANOVA on (C) age-independent and (D) age-dependent mortality rate among males using Selection (FCB and FSB) and Treatment (cold shock and no shock) as fixed factors crossed with the random block (1-5). *p*-values in bold are statistically significant.

| **Trait** | **Effect** | **SS** | **MS Num** | **DF Num** | **DF Den** | | ***F* ratio** | ***p*** |
| --- | --- | --- | --- | --- | --- | --- | --- | --- |
|  |  |  |  |  | |  |  |  |
| (C) Male | Selection (Sel) | 0.479 | 0.479 | 1.000 | | 4.021 | 2.920 | 0.162 |
| age | Treatment (Trt) | 9.979 | 9.979 | 1.000 | | 4.021 | 61.398 | **0.001** |
| independent | Block (Blk) | 8.791 | 2.198 | 4.000 | | 0.008 | 126.988 | 0.960 |
| Mortality | Sel × Trt | 0.127 | 0.127 | 1.000 | | 4.011 | 0.410 | 0.557 |
|  | Sel × Blk | 0.656 | 0.164 | 4.000 | | 4.000 | 0.531 | 0.723 |
|  | Trt × Blk | 0.649 | 0.162 | 4.000 | | 4.000 | 0.525 | 0.726 |
|  | Sel × Trt × Blk | 1.236 | 0.309 | 4.000 | | 39.00 | 1.220 | 0.318 |
|  |  |  |  |  | |  |  |  |
| (D) Male | Selection (Sel) | 3.9×10^-5^ | 3.9×10^-5^ | 1.000 | | 4.017 | 0.340 | 0.591 |
| age | Treatment (Trt) | 7.3×10^-3^ | 7.3×10^-3^ | 1.000 | | 4.030 | 112.215 | **<0.001** |
| dependent | Block (Blk) | 1.4×10^-3^ | 3.5×10^-4^ | 4.000 | | 9×10^-6^ | 3470.59 | 1.000 |
| Mortality | Sel × Trt | 1.8×10^-9^ | 1.8×10^-9^ | 1.000 | | 4.011 | 1×10^-5^ | 0.998 |
|  | Sel × Blk | 4.6×10^-4^ | 1.1×10^-4^ | 4.000 | | 4.000 | 0.638 | 0.663 |
|  | Trt × Blk | 2.6×10^-4^ | 6.4×10^-5^ | 4.000 | | 4.000 | 0.362 | 0.825 |
|  | Sel × Trt × Blk | 7.1×10^-4^ | 1.8×10^-4^ | 4.000 | | 39.00 | 1.251 | 0.305 |

**Supp. Table 4.** Effect of cold shock on lifetime fecundity (Experiment 2.2). Summary of results from lifetime fecundity data by adding day (time point) as a factor and performing repeated measures of mixed model ANOVA yielded singular fits. Therefore, this is the analysis of average fecundity only.

Model: Average.Lifetime.Fecundity~Selection*Treatment + (1|Block) + (1|Block:Selection)

> anova (fit_fec)

Type III Analysis of Variance Table with Satterthwaite's method

**Sum Sq Mean Sq Num DF Den DF F value *p***

Selection 0.002 0.002 1 4.015 0.007 0.9356

Treatment 4.164 4.164 1 47.039 23.153 **< 0.0015**

Selection:Treatment 0.027 0.027 1 47.039 0.148 0.702

ANOVA-like table for random-effects: Single term deletions

**npar logLik AIC LRT Df Pr(>Chisq)**

<none> 7 -46.545 107.09

(1 | Block) 6 -46.723 105.45 0.3569 1 0.5502

(1 | Block:Selection) 6 -55.459 122.92 17.8277 1 2.418e-05 ***

--Signif. codes: 0 ‘***’ 0.001 ‘**’ 0.01 ‘*’ 0.05 ‘.’ 0.1 ‘ ’ 1

Out put of the repeated measures" ANOVA on daywise fecundity (GOT A SINGULAR FIT)> fit_fecr = lmer(Fecundity~Selection*Treatment*Age + (1|Block) + (1|Block:Selection) + (1|Cage.id) , fecundity), boundary (singular) fit: see ? is Singular

> ANOVA (fit_fecundity)

Type III Analysis of Variance Table with Satterthwaite's method

Effect **Sum Sq Mean Sq Num DF Den DF F value *p***

Selection (Sel) 1.0 1.0 1 14.69 0.139 0.718

Treatment (Trt) 124.8 124.8 1 633.36 17.647 **<0.003**

Age or time point 5509.2 5509.2 1 633.05 779.069 **< 0.002**

Selection:Treatment 3.8 3.8 1 633.36 0.537 0.464

Selection:Age 2.9 2.9 1 633.05 0.408 0.523

Treatment:Age 80.3 80.3 1 633.05 11.350 **<0.001**

Sel:Trt:Age 3.8 3.8 1 633.05 0.3 0.466

**Supp. Table 5.** Effect of cold shock on adult mortality (Experiment 2.3). Summary of results from a four-factor mixed model ANOVA on the males and females’ mortality using selection (FCB and FSB), treatment (cold shock and no shock), and sex ( male and female) as fixed factors crossed with the random block (1-5). *p*-values in bold are statistically significant.

| **Effect** | **SS** | **MS Num** | **DF Num** | **DF Den** | ***F* ratio** | ***p*** |
| --- | --- | --- | --- | --- | --- | --- |
| Selection (Sel) | 5.712 | 5.712 | 1.000 | 4.006 | 1.106 | 0.352 |
| Sex | 69.572 | 69.572 | 1.000 | 4.003 | 6.441 | 0.064 |
| Treatment (Trt) | 170.112 | 170.112 | 1.000 | 4.004 | 22.919 | **0.009** |
| Block (Blk) | 21.332 | 5.333 | 4.000 | 0.207 | 1.116 | 0.796 |
| Sel × Sex | 0.224 | 0.224 | 1.000 | 4.029 | 0.191 | 0.684 |
| Sel × Trt | 7.316 | 7.316 | 1.000 | 4.003 | 0.575 | 0.490 |
| Sel × Blk | 20.673 | 5.168 | 4.000 | 2.487 | 0.495 | 0.749 |
| Sex × Trt | 30.148 | 30.148 | 1.000 | 4.004 | 3.683 | 0.127 |
| Sex × Blk | 43.265 | 10.816 | 4.000 | 1.734 | 1.831 | 0.406 |
| Trt × Blk | 29.723 | 7.431 | 4.000 | 5.060 | 0.425 | 0.786 |
| Sel × Sex × Trt | 0.271 | 0.271 | 1.000 | 4.010 | 0.078 | 0.793 |
| Sel × Sex × Blk | 4.680 | 1.170 | 4.000 | 4.000 | 0.338 | 0.841 |
| Sel × Trt × Blk | 50.925 | 12.731 | 4.000 | 4.000 | 3.682 | 0.117 |
| Sex × Trt × Blk | 32.778 | 8.194 | 4.000 | 4.000 | 2.370 | 0.212 |
| Sel × Sex × Trt × Blk | 13.832 | 3.458 | 4.000 | 78.000 | 1.415 | 0.237 |

**Supp. Fig. 1:** Lifetime fecundity per female (Experiment 2.2). Fecundity was measured at eleven time points once every six days with longevity. Mean fecundity per female for each population and treatment was computed for eleventime points. These results indicate that fecundity reduces with age. However, none of the other effects were significant.


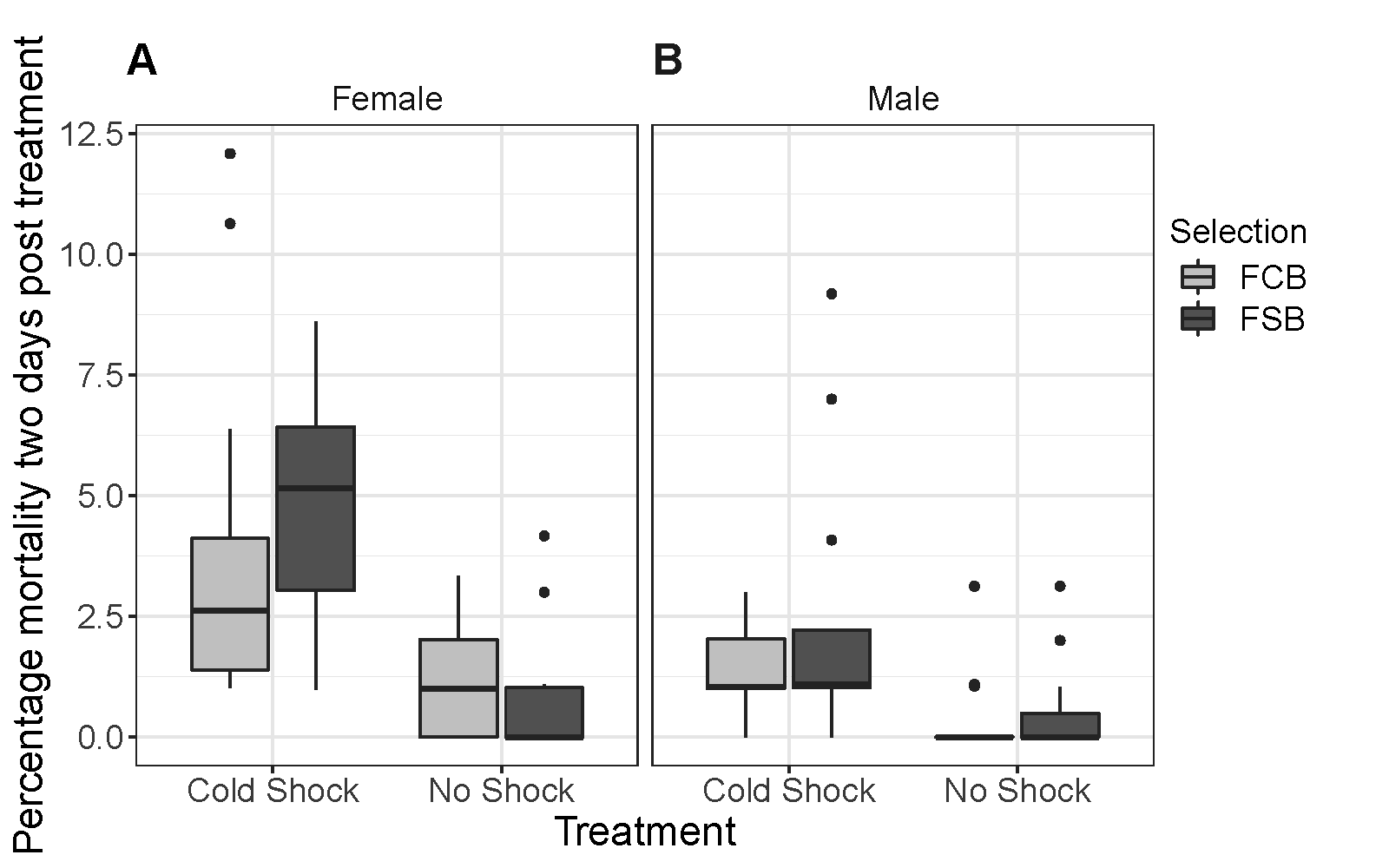


**Supp. Fig. 2:** Effect of cold shock on adult mortality (Experiment 2.3). We measured the female and male mortality 24h post cold shock. **A** Post cold shock, more females died compared to no shock treatment. However, we noticed no significant difference between FSB females and FCB females. **B** Similarly, more males died to post cold shock compared to no shock treatment. The Gray box plot represents the FCB, and the dark gray box plotrepresents the FSB populations.

**Analysis of data for normality**

**------------------------------------------------------------------------------------**

We fit linear mixed effects models (comparable to our analysis in JMP) using ‘lme4’ package in R and subsequently plotted the histograms of the residuals and tested the normality of the residuals using Shapiro test

**Models used:**

For longevity, development time, adult mortality and dry body weight:

Y~Selection*Treatment*Sex + (1|Block)

For fecundity, mating frequency, egg viability and larva to adult viability

Y~Selection*Treatment + (1|Block)

**Outputs of Shapiro Test for normality**

p- values less than 0.05 indicates non-normal distribution.

**A) Egg viability (Experiment 1)**

Shapiro-Wilk normality test

W = 0.78446, p-value = 3.263e-06

**B) Mating frequency (Experiment 1)**

Shapiro-Wilk normality test

W = 0.96024, p-value = 0.5486

**C) Mean longevity (Experiment 2.1)**

Shapiro-Wilk normality test

W = 0.99261, p-value = 0.7871

**D) Life time fecundity (Experiment 2.2)**

Shapiro-Wilk normality test

W = 0.98723, p-value = 0.7927

**E) Adult mortality (Experiment 2.3)**

Shapiro-Wilk normality test

W = 0.87036, p-value = 9.7e-09

**F) Development time (Experiment 3.1)**

Shapiro-Wilk normality test

W = 0.94044, p-value = 1.534e-11

**G) Dry body weight (Experiment 3.2)**

Shapiro-Wilk normality test

W = 0.99155, p-value = 0.02227

**H) Larva to adult survivorship (Experiment 3.3)**

Shapiro-Wilk normality test

W = 0.95092, p-value = 2.314e-06

**Data analysis using non-parametric test**

**------------------------------------------------------------------------------------**

We performed Kruskal Wallis tests followed by pairwise comparisons using Wilcoxon Rank sum exact test. The dependent variable was each of the following measurables. The independent variable was a combination of Selection and Treatment (it had four levels: FSB shocked, FSB non-shocked, FCB shocked, FCB non-shocked).

**A) Mating frequency (Experiment 1)**

Kruskal-Wallis chi-squared = 15.363, df = 3, p-value = **0.001531**

P-values forpairwise comparisons using Wilcoxon rank sum exact test

FCB Cold Shock FCB No Shock FSB Cold Shock

FCB No Shock 0.019 - -

FSB Cold Shock 0.016 0.016 -

FSB No Shock 0.600 0.019 0.016

**B) Egg Viability (Experiment 1)**

**i) 0 Hours**

Kruskal-Wallis chi-squared = 14.585, df = 3, p-value = **0.002208**

P-values for pairwise comparisons using Wilcoxon rank sum exact test

FCB Cold Shock FCB No Shock FSB Cold Shock

FCB No Shock 0.012 - -

FSB Cold Shock 1.000 0.012 -

FSB No Shock 0.012 0.415 0.012

**ii) 24 Hours**

Kruskal-Wallis chi-squared = 16.109, df = 3, p-value = **0.001077**

P-values for pairwise comparisons using Wilcoxon rank sum exact test

FCB Cold Shock FCB No Shock FSB Cold Shock

FCB No Shock 0.014 - -

FSB Cold Shock 0.014 0.014 -

FSB No Shock 0.014 0.834 0.014

**C) Mean longevity (Experiment 2)**

**i) Females**

Kruskal-Wallis chi-squared = 4.924, df = 3, p-value = 0.1774

P-values for pairwise comparisons using Wilcoxon rank sum exact test

FCB Cold Shock FCB No Shock FSB Cold Shock

FCB No Shock 0.95 - -

FSB Cold Shock 0.45 0.45 -

FSB No Shock 0.22 0.22 0.45

**ii) Males**

Kruskal-Wallis chi-squared = 3.0499, df = 3, p-value = 0.384

P-values for pairwise comparisons using Wilcoxon rank sum exact test

FCB Cold Shock FCB No Shock FSB Cold Shock

FCB No Shock 0.58 - -

FSB Cold Shock 0.93 0.45 -

FSB No Shock 0.93 0.45 0.93

**D) Lifetime fecundity (Experiment 2.2)**

Kruskal-Wallis chi-squared = 8.4896, df = 3, p-value = 0.03691

P-values for pairwise comparisons using Wilcoxon rank sum exact test

FCB Cold Shock FCB No Shock FSB Cold Shock

FCB No Shock 0.123 - -

FSB Cold Shock 0.477 0.022 -

FSB No Shock 0.477 0.477 0.232

**E) Adult mortality (Experiment 2.3)**

**i) Females**

Kruskal-Wallis chi-squared = 28.804, df = 3, p-value = **2.462e-06**

P-values for pairwise comparisons using Wilcoxon rank sum test with continuity correction.

FCB Cold Shock FCB No Shock FSB Cold Shock

FCB No Shock 0.00419 - -

FSB Cold Shock 0.20298 0.00022 -

FSB No Shock 0.00065 0.50580 0.00022

**ii) Males**

Kruskal-Wallis chi-squared = 16.901, df = 3, p-value = **0.0007407**

P-values for pairwise comparisons using Wilcoxon rank sum test with continuity correction

FCB Cold Shock FCB No Shock FSB Cold Shock

FCB No Shock 0.0158 - -

FSB Cold Shock 0.3510 0.0088 -

FSB No Shock 0.0162 0.7583 0.0088

**F) Development time (Experiment 3.1)**

**i) Females**

Kruskal-Wallis chi-squared = 28.534, df = 3, p-value = **2.805e-06**

P-values for pairwise comparisons using Wilcoxon rank sum test with continuity correction.

FCB Cold Shock FCB No Shock FSB Cold Shock

FCB No Shock 0.4332 - -

FSB Cold Shock 9.5e-07 0.0096 -

FSB No Shock 0.0004 0.0120 0.7055

**ii) Males**

Kruskal-Wallis chi-squared = 48.968, df = 3, p-value = **1.325e-10**

P-values for pairwise comparisons using Wilcoxon rank sum test with continuity correction.

FCB Cold Shock FCB No Shock FSB Cold Shock

FCB No Shock 0.3280 - -

FSB Cold Shock 5.2e-07 2.2e-09 -

FSB No Shock 0.0026 7.4e-05 0.1318

**G) Dry body weight (Experiment 3.2)**

**i) Females**

Kruskal-Wallis chi-squared = 13.968, df = 3, p-value = **0.002948**

P-values for pairwise comparisons using Wilcoxon rank sum test with continuity correction.

FCB Cold Shock FCB No Shock FSB Cold Shock

FCB No Shock 0.9752 - -

FSB Cold Shock 0.0752 0.0752 -

FSB No Shock 0.0075 0.0075 0.4009

**ii) Males**

Kruskal-Wallis chi-squared = 3.4927, df = 3, p-value = 0.3217

P-values for pairwise comparisons using Wilcoxon rank sum test with continuity correction.

FCB Cold Shock FCB No Shock FSB Cold Shock

FCB No Shock 0.54 - -

FSB Cold Shock 0.54 0.69 -

FSB No Shock 0.51 0.69 0.54

**H) Larva to adult survivorship (Experiment 3.3)**

Kruskal-Wallis chi-squared = 6.838, df = 3, p-value = 0.07724

P-values for pairwise comparisons using Wilcoxon rank sum test with continuity correction

FCB Cold shock FCB No shock FSB Cold shock

FCB No shock 0.087 - -

FSB Cold shock 0.694 0.139 -

FSB No shock 0.694 0.139 0.891

BH

**Analysis of data using Generalized Linear Model (GLM)**

**------------------------------------------------------------------------------------**

**A) Egg viability (Experiment 1)**

Mixed model anova with selection, treatment and period as fixed factors and block as random.

Model: PropV~Selection*Treatment*Period + (1|Block)

Type III Analysis of Variance Table with Satterthwaite's method

**Effect Sum Sq Mean Sq NumDF DenDF F value *p***

Selection(Sel) 0.0897 0.0897 1 28 34.214 **2.747e-06 *****

Treatment (Trt) 5.1296 5.1296 1 28 1956.441 **< 2.2e-16 *****

Period(Per) 0.5778 0.5778 1 28 220.370 **8.462e-15 *****

Sel:Trt 0.0912 0.0912 1 28 34.789 **2.407e-06 *****

Sel:Per 0.0904 0.0904 1 28 34.480 **2.584e-06 *****

Trt:Per 0.5511 0.5511 1 28 210.190 **1.524e-14 *****

Sel:Trt:Per 0.0904 0.0904 1 28 34.489 **2.579e-06 *****

--Signif. codes: 0 ‘***’ 0.001 ‘**’ 0.01 ‘*’ 0.05 ‘.’ 0.1 ‘ ’ 1

ANOVA-like table for random-effects: Single term deletions

npar logLik AIC LRT Df Pr(>Chisq)

<none> 10 41.992 -63.983

(1 | Block) 9 41.582 -65.164 0.81956 1 0.3653

**B) Mean longevity (Experiment 2)**

Mixed model anova for mean longevity with selection, treatment and sex as fixed factors and block, block*selection, cage.id as random factors.

Model: Mean~Selection*Treatment*Sex + (1|Block) + (1|Block:Selection) + (1|Cage.id)

Type III Analysis of Variance Table with Satterthwaite's method

**Sum Sq Mean Sq NumDF DenDF F value *p***

Selection 43.66 43.66 1 4.106 3.7630 0.1226

Treatment 3.79 3.79 1 47.295 0.3266 0.5704

Sex 1293.62 1293.62 1 55.000 111.5024 **7.657e-15 *****

Selection:Treatment 6.03 6.03 1 47.295 0.5194 0.4746

Selection:Sex 1.21 1.21 1 55.000 0.1044 0.7478

Treatment:Sex 12.18 12.18 1 55.000 1.0501 0.3100

Selection:Treatment:Sex 3.09 3.09 1 55.000 0.2660 0.6081

--Signif. codes: 0 ‘***’ 0.001 ‘**’ 0.01 ‘*’ 0.05 ‘.’ 0.1 ‘ ’ 1

ANOVA-like table for random-effects: Single term deletions

npar logLik AIC LRT Df Pr(>Chisq)

<none> 12 -315.46 654.93

(1 | Block) 11 -316.11 654.22 1.29897 1 0.2544

(1 | Block:Selection) 11 -315.50 653.00 0.07893 1 0.7788

(1 | Cage.id) 11 -316.43 654.85 1.92459 1 0.1654

**D) Adult mortality (Experiment 2.3)**

We fit a logistic regression on the status of the flies (1 = alive, 0 = dead) 2 days after treatment.

Selection, treatment and sex were fixed factors. Block and cage.id were random factors.

Model: Status~Selection*Treatment*Sex + (1|Block) + (1|Cage.id)

Random effects:

Groups Name Variance Std.Dev.

Cage.id (Intercept) 2.684e-01 0.5180506

Block (Intercept) 6.858e-08 0.0002619

Number of obs: 11378, groups: Cage.id, 59; Block, 5

| **Fixed effects:** | **Estimate** | **Std. Error** | **z value** | ***p*** |
| --- | --- | --- | --- | --- |
| (Intercept) | 3.3333 | 0.20422 | 16.322 | **< 2e-16 ***** |
| Selection (Sel) FSB | -0.25042 | 0.27252 | -0.919 | 0.35814 |
| Treatment (Trt) No Shock | 1.35906 | 0.35581 | 3.82 | **0.000134 ***** |
| Sex Male | 1.10795 | 0.27407 | 4.043 | **5.29e-05 ***** |
| Sele FSB:Trt No Shock | 0.52386 | 0.51425 | 1.019 | 0.30835 |
| Sel FSB:Sex Male | -0.32794 | 0.3469 | -0.945 | 0.344492 |
| Trt No Shock:Sex Male | -0.02678 | 0.58075 | -0.046 | 0.963226 |
| Sel FSB:Trt No Shock:Sex Male | -0.23011 | 0.77811 | -0.296 | 0.767434 |

--Signif. codes: 0 ‘***’ 0.001 ‘**’ 0.01 ‘*’ 0.05 ‘.’ 0.1 ‘ ’ 1

Correlation of Fixed Effects:

| **(Intr) SFSB TNS Sex Male SFSB:TNS SFSB:SM TNS:SM SFSB:TNS:SM** | |
| --- | --- |
| **Selection(S)FSB -0.736** |  |
| **Treatment NoShock(TNS) -0.553 0.418** | |
| **Sex Male -0.355 0.267 0.205** | |
| **SFSB:TNS 0.397 -0.531 -0.689 -0.141** | |
| **SFSB:SM 0.282 -0.367 -0.162 -0.790 0.194** | |
| **TNS:SM 0.168 -0.126 -0.416 -0.472 0.287 0.373** | |
| **SFSB:TNS:SM -0.125 0.163 0.310 0.352 -0.457 -0.446 -0.746** | |

Analysis of Deviance Table (Type II Wald chisquare tests)

Response: Status

**Chisq Df Pr(>Chisq)**

Selection(Sel) 0.9636 1 0.3263

Treatment (Trt) 47.1036 1 **6.733e-12 *****

Sex 33.8259 1 **6.027e-09 *****

Sel:Trt 0.9866 1 0.3206

Sel:Sex 1.4481 1 0.2288

Trt:Sex 0.1608 1 0.6885

Sel:Trt:Sex 0.0875 1 0.7674

--Signif. codes: 0 ‘***’ 0.001 ‘**’ 0.01 ‘*’ 0.05 ‘.’ 0.1 ‘ ’ 1

**E) Development Time (Experiment 3.1)**

This is a mixed model ANOVA with selection treatment and sex as fixed factors and blocks as random factor.

Model: lmer(Mean.DT~Selection*Treatment*Sex + (1|Block)

> anova(fit_dt)

Type III Analysis of Variance Table with Satterthwaite's method

**Sum Sq Mean Sq NumDF DenDF F value *p***

Selection 1288.2 1288.2 1 386.01 74.4369 **< 2e-16 *****

Treatment 26.5 26.5 1 386.00 1.5325 0.21649

Sex 3660.1 3660.1 1 385.99 211.4964 **< 2e-16 *****

Selection:Treatment 0.0 0.0 1 386.01 0.0004 0.98403

Selection:Sex 110.4 110.4 1 386.00 6.3778 **0.01196 ***

Treatment:Sex 69.3 69.3 1 385.99 4.0031 **0.04612 ***

Selection:Treatment:Sex 8.9 8.9 1 386.00 0.5153 0.47329

--Signif. codes: 0 ‘***’ 0.001 ‘**’ 0.01 ‘*’ 0.05 ‘.’ 0.1 ‘ ’ 1

ANOVA-like table for random-effects: Single term deletions

npar logLik AIC LRT Df Pr(>Chisq)

<none> 10 -1128.1 2276.3

(1 | Block) 9 -1132.5 2283.0 8.7516 1 **0.003093 ****

--Signif. codes: 0 ‘***’ 0.001 ‘**’ 0.01 ‘*’ 0.05 ‘.’ 0.1 ‘ ’ 1

**F) Larva to adult survivorship (Experiment 3.3)**

**Logsitic regression for larva to adult survivorship.** We fit a logitic regression for larva to adult survivorship. Selection, treatment were fixed factors. Block and vial id were considered as random factors.

Model: Status ~ Selection * Treatment + (1 | Block) + (1 | Vial.Id)

Random effects:

Groups Name Variance Std.Dev.

Vial.Id (Intercept) 1.1145 1.0557

Block (Intercept) 0.4055 0.6368

Number of obs: 5932, groups: Vial.Id, 200; Block, 5

Fixed effects:

**Estimate Std. Error z value *p***

(Intercept) 4.3650 0.3861 11.305 **< 2e-16 *****

Selection FSB -0.1392 0.3304 -0.421 0.67345

Treatment No shock -0.9123 0.3122 -2.922 **0.00348 ****

Selection FSB:Treatment No shock 0.7033 0.4457 1.578 0.11458

--Signif. codes: 0 ‘***’ 0.001 ‘**’ 0.01 ‘*’ 0.05 ‘.’ 0.1 ‘ ’ 1

Correlation of Fixed Effects:

(Intr) Sel FSB Trt No Shock

Selection (Sel)FSB -0.446

Treatment (Trt)No shock -0.496 0.547

SeltFSB:Trt No Shock 0.344 -0.742 -0.699

> ANOVA(fit_survival larva to adult)

Analysis of Deviance Table (Type II Wald chisquare tests)

Response: Status

Chisq Df Pr(>Chisq)

Selection 1.2535 1 0.26288

Treatment 6.4721 1 **0.01096 ***

Selection:Treatment 2.4898 1 0.11458

--Signif. codes: 0 ‘***’ 0.001 ‘**’ 0.01 ‘*’ 0.05 ‘.’ 0.1 ‘ ’ 1
